# Spatially-Resolved Network Dynamics of Poly(vinyl alcohol) Gels Measured with Dynamic Small Angle Light Scattering

**DOI:** 10.3390/gels8070394

**Published:** 2022-06-22

**Authors:** Sujata Dhakal, Zehao Chen, Daniel Estrin, Svetlana Morozova

**Affiliations:** 1 Department of Macromolecular Science and Engineering, Case Western Reserve University, Cleveland, OH 44106, USA; sxd729@case.edu (S.D.); dye3@case.edu (D.E.); 2 Department of Chemical and Biomolecular Engineering, Case Western Reserve University, Cleveland, OH 44106, USA; zxc318@case.edu

**Keywords:** gel dynamics, poly(vinyl alcohol), spatially-resolved

## Abstract

Hydrogels are cross-linked polymer networks swollen in water. The large solvent content enables hydrogels to have unique physical properties and allows them to be used in diverse applications such as tissue engineering, drug delivery, and absorbents. Gel properties are linked to internal dynamics. While bulk gel dynamics have been studied extensively, how gel networks respond locally to deformation has yet to be understood. Here, poly(vinyl alcohol) (PVA) gels have been stretched to study the effects of deformation on gel dynamics parallel and perpendicular to the stretching direction using dynamic small angle light scattering (DSALS). The implementation of DSALS is described and compared to traditional DLS for PVA gels with different crosslink densities, ranging from 0.75–2%. Despite the orders of magnitude difference in the scattering vector, *q*, range of the techniques, the dynamics match, and the apparent elastic diffusion coefficient, *D*_A_ increases linearly with the crosslink density for unstretched gels at a constant 2 wt% concentration. We observe that the elastic motion depends on the direction of stretch, decreasing perpendicular to stretching and increasing at parallel direction. Using DSALS can therefore be an effective tool to evaluate local hydrogel response to deformation.

## 1. Introduction

Dynamic light scattering (DLS) is a well-established technique used to study the dynamics and elasticity of particles, polymers, and gels [1,2,3]. In particular, it has been used as a non-invasive way to measure the elastic properties of soft materials such as poly(vinyl alcohol) (PVA) hydrogels [4,5]. PVA gels are widely used in biomedical applications, including tissue engineering and drug delivery [6,7,8,9,10,11]. The mechanical properties and phase separation of PVA gels have been studied extensively using scattering techniques [4,5,12,13,14]. However, DLS is complicated by the natural heterogeneous nature of gels, which often results in fluctuating and nonfluctuating contributions to the signal [12,15,16,17]. In addition, it is difficult to measure the material response to deformation or any resulting dynamic anisotropy using DLS. Here, we discuss how dynamic small angle light scattering (DSALS) can provide information on spatial variations in dynamics for stretched and unstretched PVA gels.

Benchtop small angle light scattering (SALS) instruments have been implemented for a long time to measure 2D light scattering patterns from complex fluids, gels, and biological systems and the temporal evolution of those patterns [18,19,20,21,22,23,24,25,26]. This technique is especially useful for observing structure on ~0.5–10 µm size scales. SALS instruments typically follow established optical paths and are widely versatile. As a result, SALS has been integrated into microscopes, rheometers, flow cells, and microfluidics [24,25,27]. It is also possible to simultaneously measure the scattering patterns and the transmitted intensity of a sample using a modified SALS setup [28]. The scattering angles, *θ*, associated with SALS is <10°, which corresponds to scattering vector, *q =* $\frac{4\pi n}{\lambda }\sin(\theta /2)$, values of 0.00035–0.035 nm^−1^, where *n* is the index of refraction of the sample, and *λ* is the laser wavelength.

The intensity collected at small angles has also been autocorrelated to evaluate diffusion and viscoelastic properties of the sample, much like in a traditional, wide angle Dynamic Light Scattering (DLS) instrument or in the recently-developed X-ray Photon Correlation Spectroscopy (XPCS) (Table 1). Previous implementations of this technique have relied on a light scattering microscope design [29], or autocorrelation of small angle light scattering intensities [20]. These setups have been used to determine the dynamics of nanoparticles in oil and nonergodic systems such as agarose. A charged coupling device (CCD) detector has also been used to obtain intensities from a small angle scattering pattern, which are then autocorrelated to determine dynamics at many angles simultaneously [20]. In their study, Cipelletti and Weitz [20] note that such a setup can be used to obtain the ensemble average directly to image nonergodic systems such as gels. Recently, the autocorrelation of a speckle pattern in a CMOS camera was used to measure blood flow dynamics [30]. In XPCS, like in DLS with visible light, fluctuations in the coherent scattering intensity from a material are related to dynamics, but over significantly shorter distances due to a difference in the scattering vector *q* (Table 1) [31]. These techniques have been used to determine the dynamics of surfactants, gels, block copolymer phases, and colloidal suspensions [31,32]. Recently, XPCS has also been used to study local material response to deformation and flow. Ehrburger-Dolle et al. used probe particles to determine dynamic anisotropy in a stretched elastomer. They find that particle interactions with the polymer complicate the interpretation of network dynamics [33]. The advantage of DLS, XPCS, and DSALS techniques is that the structural resolution is not necessary to resolve dynamics at a particular *q* vector. Because of this, DLS and DSALS, which are both optical techniques, have been used to observe dynamics of nanometer objects.

**Figure 1:**
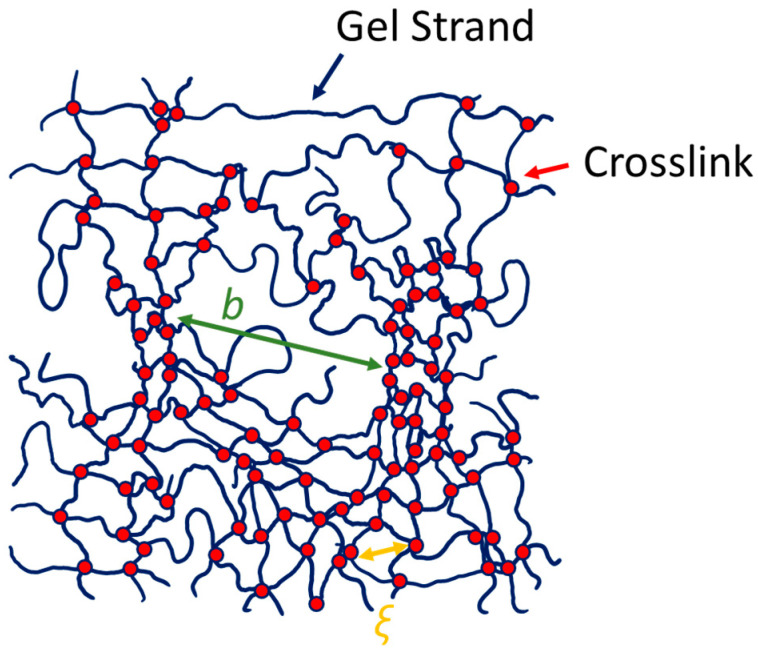
A schematic representation of a polymer gel, in which the gel strands (blue) are covalently linked by crosslinks (red). Since the crosslinking is random, the network topology can be described by an average mesh size, *ξ*, and regions of higher crosslinking density, separated by a distance *b*.

Static scattering of gels has been shown to depend on the network topology for both stretched and unstretched gels. Small angle neutron techniques have been used to evaluate the heterogeneous nature of unstretched, randomly crosslinked gels. Due to the random nature of the crosslinking chemistry, typically, there are areas of high crosslink density, separated by a length-scale *b*, surrounded by areas of average crosslink density, characterized by the average polymer spacing, the mesh size, *ξ* (Figure 1) [3,14,34,35,36]. The heterogeneous nature of gels has been shown to contribute to both static and dynamic measurements. PVA gels typically show a “slow” and a “fast” relaxation mode, and have been shown to be more nonergodic than poly(acrylic acid) gels. Typically, there is a strongly varying degree of nonergodicity [12]. Shibayama et al. [37] have evaluated 2D small angle neutron scattering patterns of gels after stretching. They find that any anisotropy in the scattering pattern is correlated with heterogeneity, and that ‘ideal’ tetra-arm polyethylene glycol gels only showed a slight change in the scattering pattern, and an emergence of an intensity upturn at low *q*. In contrast, clay-filled gels [38] and poly(N-isopropylacrylamide)/acrylic acid gels showed a characteristic ‘butterfly’ pattern after deformation [35,39]. Few dynamic studies have been performed to determine any dynamic anisotropy during deformation. One study by Takebe et al. [40] showed that the apparent elastic diffusion coefficient, *D*_A_, determined from DLS, is slower perpendicular to the direction of stretching deformation and faster in the parallel direction.

We have determined the spatially-resolved dynamics of poly(vinyl alcohol) (PVA) covalently-crosslinked gels by autocorrelating intensity fluctuations in a 2D SALS scattering pattern. The implementation of this method, termed DSALS, is described and compared to DLS by comparing the PVA network dynamics for different crosslink densities, ranging from 0.75–2%, for a constant 2 wt% gel concentration. In this technique, intensity correlations of multiple *q* vectors in a 2D scattering pattern makes direct ensemble averaging possible. This enables the measurement of the dynamic anisotropy in PVA gels as a function of stretching.

## 2. Results and Discussion

We have used Dynamic Small Angle Light Scattering (DSALS) to determine PVA gel network dynamics as a function of the scattering angle *θ* and the spatial azimuthal angle, *ψ*. The obtained data for PVA gels with different crosslink densities are compared and contrasted to a well-established technique, DLS, conforming the results. Since DSALS can provide information about spatial variation of dynamics within the gel, we use this technique to determine how the gel network motion varies in PVA gels as a function of deformation, by comparing the intensity correlations in the direction of stretching and perpendicular to the direction of stretching.

### 2.1. Unstretched Gels

**Figure 2:**
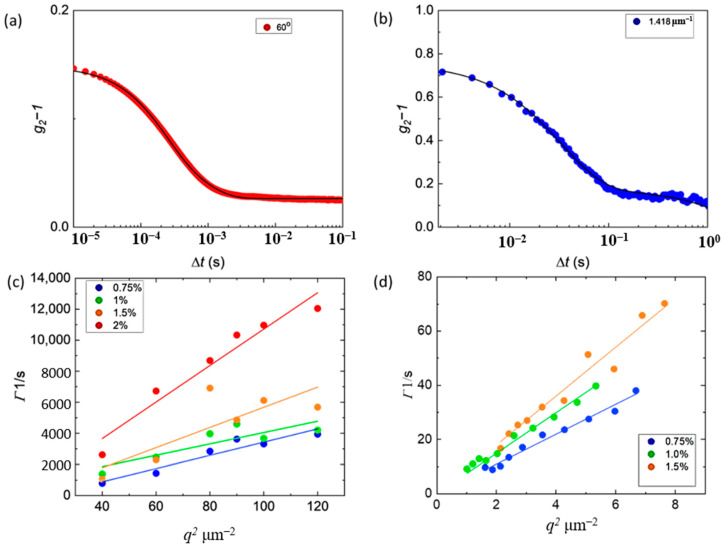
PVA dynamics determined from DSALS and DLS as a function of crosslinking density. (**a**) DLS intensity correlations at *θ* = 60°. The black line is a double exponential fit to the data. (**b**) DSALS intensity correlations at *q* = 1.423 µm^−1^. The black line is a double exponential fit to the data. (**c**) The dominant fast relaxation rate *Γ* determined from the exponential fit vs. *q*^2^ as a function of crosslink density determined from DLS. (**d**) The dominant fast relaxation rate *Γ* determined from the exponential fit vs. *q*^2^ as a function of crosslink density determined from DSALS.

To demonstrate the validity of the DSALS design, we compare the *q*-dependent autocorrelations from PVA gels with different crosslink densities to DLS. For both experiments, PVA gels were prepared in the instrument sample geometry and allowed to gel overnight (See Section 4 below). The scattered intensity was autocorrelated and sample autocorrelation functions are shown in Figure 2a,b. The correlation functions were fit to a sum of two exponential decays to determine the dominant relaxation rate in the system, *Γ*. *Γ* versus *q*^2^ plots are shown in Figure 2c,d as a function of crosslink %. The apparent elastic diffusion coefficient *D*_A_ is determined from the slope. For a 2 wt% PVA gel with 0.75% crosslinking, the apparent *D*_A_ determined from DSALS is 5.5 ± 0.2 µm^2^/s and 7 ± 1 µm^2^/s determined from DLS. We observe that the faster relaxation mode is the most dominant for gels with lower crosslinking densities. As the crosslinking increases, *D*_A_ increases, the sample becomes less transparent and the slower relaxations due to the static field from inhomogeneities start to dominate (See Supplementary Information (Figures S1–S4)). The slower relaxations are present in DLS as well, but are not fully resolved, and show up as a drift in the total intensity over time. This is consistent with previous observations of PVA gel dynamics determined with DLS [12]. In general, the PVA gel dynamics observed in DLS and DSALS are very similar.

**Figure 3:**
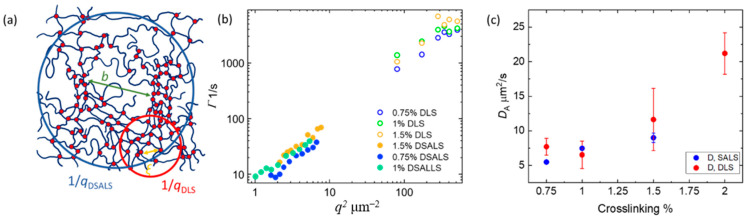
Comparison of PVA gel dynamics determined from DLS and DSALS. (**a**) A schematic illustrating the difference in *q*-vectors for DLS and DSALS where an average mesh size is, *ξ*, and the distance between the regions of higher crosslinks, *b*. (**b**) The fast relaxation rates plotted across the whole *q*^2^ range from both techniques. (**c**) The apparent diffusion coefficient *D*_A_ as a function of crosslinking %. The values from DLS and DSALS are within error. Error bars represent standard error from the linear fit to *Γ* vs. *q*^2^ trends.

The differences between the two techniques may be explained by the drastic *q*-range differences. While the *q* range in DLS is 0.012–0.22 nm^−1^, the range for DSALS is 0.001–0.0025 nm^−1^; therefore, as illustrated in Figure 3a, any inhomogeneities on the 3–6 µm scale will contribute to the overall dynamics. This is clearly observed when comparing the typical correlation functions collected from DLS and DSALS. While in DLS, there is often only one resolved decay, and more often than not, two decays are fully resolved in the DSALS intensity correlations. Despite the differences in the *q* range, the *Γ* versus *q*^2^ data can be plotted across the whole *q*-range (Figure 3b), indicating that the two techniques measure similar dynamics for all gels studied.

With both DLS and DSALS, there is a clear trend between *D*_A_ and crosslinking density. As the crosslinking increases from 0.75% to 2%, *D*_A_ increases linearly from 5.5–7 µm^2^/s to 20 µm^2^/s. The linear trend is expected for a constant polymer concentration in the gel, since the friction coefficient is unchanged, and therefore the only difference between gel systems is the modulus, *G*_l_, which is expected to increase linearly with crosslinking density, if all else is equal.

**Figure 4:**
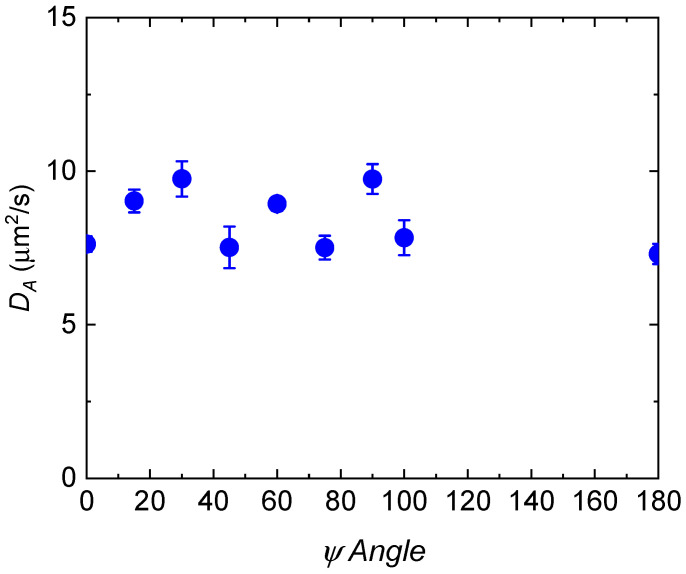
Apparent elastic diffusion coefficient as a function of the spatial azimuthal angle for an unstretched 2 wt% 1% crosslink PVA gel.

The advantage to DSALS is that all *q* vectors are simultaneously temporally resolved and spatial information within the gel is obtained. Hence, it is possible to quantify the ensemble average in addition to the time average within the sample using the spatial information. To quantify the spatial variation within the gel, *D*_A_ was determined as a function of the spatial azimuthal angle, *ψ* (Figure 4) for a 2 wt% 1% crosslinked PVA gel. The range of the values for all different angles is from 7.5–9.5 µm^2^/s, with a mean of 8 µm^2^/s, and a standard deviation of 1 µm^2^/s. As expected, there is some spatial variation within the gel, but the *D*_A_ values are similar across the whole sample, which is consistent with an unperturbed and isotropic gel. Therefore, this optical setup can be used to understand the µm-scale variations in dynamics for anisotropic gel samples, for example, for gels as a function of deformation.

### 2.2. Stretched Gels

The dynamics of stretched 2 wt% 1% crosslink PVA gels were determined as a function of the spatial azimuthal angle, *ψ*, in the direction of stretching and perpendicular to the direction of stretching. Little is known about how the random network motion changes due to gel deformation. One study by Takebe et al. [40] looked at the DLS-determined intensity correlations for polyacrylamide gels as a function of stretching. To determine dynamic variations spatially within the gel, they rotated the sample relative to the incident beam. They find that as a function of stretching, *D*_A_ parallel and perpendicular to the stretching direction diverge, increasing in the parallel direction and decreasing in the perpendicular direction. Recently, many experiments have focused on understanding the static scattering intensity as a function of the azimuthal angle during deformation. These experiments conclude that for small angle scattering, the static inhomogeneities dominate the anisotropy in the scattering pattern [39,41].

**Figure 5:**
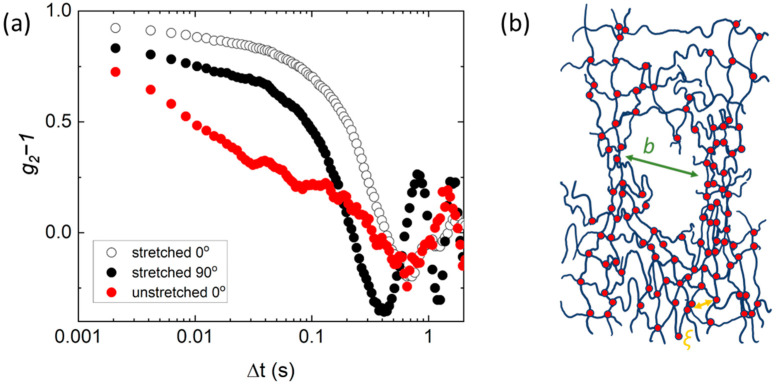
(**a**) Intensity autocorrelation function at *q* = 2.245 µm^−1^ for a stretched gel at *ψ* = 0° (perpendicular to stretching) and 90° (parallel to stretching) compared to an unstretched gel at 0°. (**b**) A schematic representation of local gel deformation at ~3 µm length scale.

To study dynamics as a function of stretching, PVA was gelled in a silicone mold that was secured in a home-made sample geometry (See Supplementary Information) and stretched 1.3 times. The correlation functions at *q* = 2.245 µm^−1^ are compared in Figure 5a parallel to the stretching direction (*ψ* = 90°) and perpendicular to the stretching direction (*ψ* = 0°). For reference, the correlation function at *ψ* = 0° for an unstretched gel is included in the Figure 5a. We observe that the slower relaxation mode increases in intensity for the stretched gel in all direction, and there is a clear anisotropy in the dynamics. Parallel to the stretching direction, the dynamics are considerably faster relative to both the unstretched gel and the direction perpendicular to stretching, as indicated by the shift of the correlation function to shorter time scales. We also observe oscillations in the correlation function, especially in the *ψ* = 90° direction, which makes fitting to determine the exact dynamics difficult. This behavior is observed for all *q* vectors analyzed (See Supplementary Information). These observations are also observed in XPCS experiments for deformed materials [31].

The observed increase in the slower mode intensity and anisotropy is consistent with previous observations of small angle scattering on deformed gels [38]. As the gel is stretched, the deformation is likely supported by areas of higher crosslink density, separated by average length *b* (Figure 5b). This results in anisotropy of the “inhomogeneities” in the gel, and possibly void formation or orientation, which leads to a higher overall contribution to the correlation function. Although these observations are consistent with previous publications, more work is needed to fully understand how gels respond locally to deformation. We believe that the DSALS technique coupled with more structural characterization could lead to an unprecedented understanding of this behavior.

## 3. Conclusions

We have incorporated dynamic capabilities into a traditional SALS setup to determine spatially-resolved network fluctuations in poly(vinyl alcohol) gels as a function of crosslink density and deformation. DSALS is an effective method to determine gel dynamics as a function of the scattering angle *θ* and the spatial azimuthal angle, *ψ*. The apparent diffusion coefficient, *D*_A_, obtained from DSALS is within error of a well-established technique, DLS which confirms the adequacy of the technique. Using both DLS and DSALS, we find that *D*_A_ increases linearly from 5.5–7 µm^2^/s to 20 µm^2^/s as a function of crosslink density for gels at a constant concentration, as expected. DSALS also provides information about the spatial variation of the dynamics for PVA gels. For unstretched gels, the spatial variation is related to the spatial inhomogeneity and nonergodicity of the networks. As the gels are stretched, there is an apparent anisotropy in the dynamics. The slower relaxation mode increases in intensity for the stretched gel in all direction. Moreover, the dynamics are faster parallel to the stretching direction and slower in the perpendicular directions. These results align with previous works in the field. The oscillation pattern obtained for the stretched gel made it difficult to fit the intensity correlation function and further study is necessary. However, the information obtained from the spatial variation of dynamics for the unstretched PVA gel system prove the efficacy of DSALS technique. We conclude that DSALS is a powerful technique, which enables studying the local anisotropy of soft materials.

## 4. Materials and Methods

### 4.1. Materials

All reagents, including the poly(vinyl alcohol) (PVA) (*M*_w_~145,000), hydrogen chloride, and glutaraldehyde, were purchased from Sigma Aldrich, (St. Louis, MO, USA )and used without further purification.

### 4.2. Dynamic Light Scattering

The fluctuations of the scattered light from a gel are related to the dynamic, random motion of the network. Dynamic Light Scattering (DLS) is a powerful tool that has been used to investigate network motion and elasticity [3,12,16,42,43,44,45,46,47,48,49,50]. In the experiment, the PVA gel was prepared by first dissolving 2 wt% PVA in 0.1 M HCl solution in a scintillation vial. The sample was stirred on a hot plate at 100 °C for full dissolution. Then, the sample was transferred to a cleaned DLS culture tube through a 0.45 µm PES filter and 0.75–3 molar% glutaraldehyde was added. The sample solution was allowed to gel overnight. The culture tube was then placed in the sample holder decalin vat in the BI-200SM Research Goniometer System and illuminated with a 30 mW laser. The scattered light was autocorrelated as a function of scattering angle, *θ*, from 30–120 °C.

First derived by Tanaka et al. [51], in a DLS experiment for a perfect network, the autocorrelated intensity decays as:

(1) $$g_{2}(q,\Delta t)-1=\beta \exp\left(\frac{-2G_{l}q^{2}\Delta t}{f}\right)$$

Therefore, the decay rate *Γ* is directly related to the longitudinal compression modulus of the network, $G_{l}$, and inversely related to friction coefficient of the polymer in the solvent, *f*:

(2) $$\Gamma =G_{l}q^{2}/f$$

How this decay rate is related to the scattering vector *q* can be termed the elastic “diffusion coefficient”, *D*_e_ = *G*_l_/*f*, and is related to the network motion. The friction coefficient, *f*, depends on the volume fraction of the network and the viscosity of the surrounding fluid, and can be estimated as $f\propto \eta \varphi^{1.5}$ [52].

For gels, the interpretation of scattering patterns is complicated because the materials are non-ergodic due to trapped inhomogeneities. This is illustrated in Figure 1. Typically, crosslinking reactions result in areas of higher crosslink density, separated by a large average distance *b*, and areas of average crosslink density, corresponding to a mesh size *ξ*. The trapped inhomogeneities manifest in a static scattered electric field, ${\langle I\rangle }_{s}$ in addition to the field that results from the gel network fluctuations, ${\langle I\rangle }_{f}$ [3,12,16,17,47,48,50,53,54]. The interpretation of the intensity correlations, therefore, needs to account for both ${\langle I\rangle }_{s}$ and ${\langle I\rangle }_{f}$. One way to do this is through the partial heterodyne method. In this method, the normalized intensity correlation function *g*_2_^−1^ is interpreted as a function of $X=\frac{{\langle I\rangle }_{f}}{{\langle I\rangle }_{s}+{\langle I\rangle }_{f}}$ and the normalized electric field correlation function, *g*_1_ [16,17]:

(3) $$g_{2}(q,\Delta t)-1=X^{2}{\left[g_{1}(q,\Delta t)\right]}^{2}+2X(1-X)g_{1}(\Delta t)$$

where the first term is the homodyne term, and the second term is the heterodyne term. It follows that the partial heterodyne apparent diffusion coefficient, *D*_A_ is equal to [17]:

(4) $$D_{A}=D_{e}/(2-X)$$

The value of *X* is determined from the amplitude of the normalized intensity autocorrelations, $\sigma^{2}=g_{2}(q,0)-1$, and the condition that $g_{1}(q,0)=1$. In this way, scattering from non-ergodic systems such as gels can be difficult to interpret without doing the ensemble average manually. One way this has been achieved is by physically rotating the sample during imaging However, the physical rotation would be unnecessary if the spatial information is given in the experiment.

### 4.3. Optical Design

**Figure 6:**
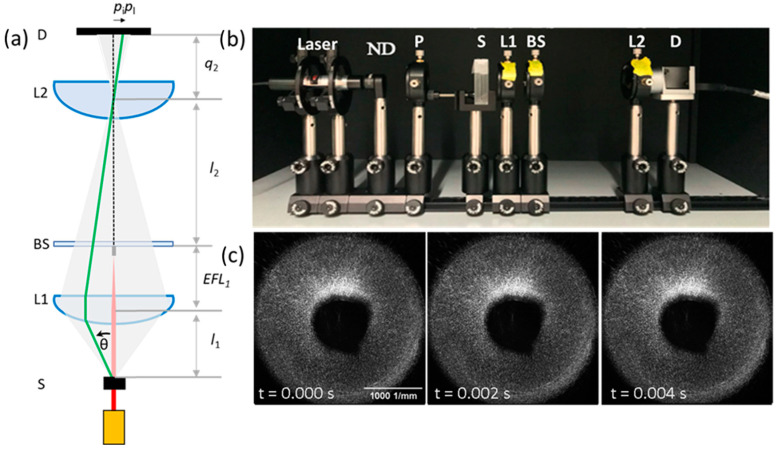
DSALS optical design. (**a**) Ray diagram of scattered light from the sample S, at an angle *θ*, as it is focused by L1 through a beamstop BS, through L2 to a CMOS detector D. (**b**) A photo of the benchtop setup. (**c**) Typical scattering patterns at the detector, at a frame rate of 480 fps, the intensity of which can be autocorrelated as a function of *θ* and the spatial azimuthal angle *ψ*.

Traditionally, DLS experiments have been performed using a detector on a goniometer and an autocorrelator, which autocorrelates the scattered intensities as a function of *q* one angle at a time. With an addition of a high-speed CMOS camera, a Small Angle Light Scattering (SALS) setup can be designed to study the dynamics of polymeric systems for a range of q values simultaneously, which allows obtaining the ensemble average directly [20,55]. A benchtop SALS setup is shown in Figure 6. A laser shines through the sample (S), which causes the light to scatter. The scattered light then reaches a lens (L1: Edmund Optics–NT67-245) with a high numerical aperture (N.A. = 0.85) that captures light up to 58° scattered angle and refocuses it, making sure that the scattering information stays within the confines of the system. The optimal position for the sample is a focal length away, or slightly farther, from L1. If placed closer, it is impossible to focus the scattered light to a point. The light then passes through a beam stop (BS), also located a focal length away from L1, which blocks the incident intensity of the initial laser beam. This is to prevent the high intensity of the laser from interfering with the lower intensity of the scattered light. The refocused light then approaches another lens (L2: Thorlabs-LA1951A) which expands the scattered light and projects it onto the camera (D: Basler acA800-510 um) placed a focal length away from L2. The overall *q* range for this set-up depends on the proximity of the sample to L1, the distance between L1 and L2, and the size of the detector. The angle information is coded into the radial pixel index vector based on the geometry. For the setup described above,

(5) $$\theta =\frac{p_{i}p_{l}}{q_{2}l_{1}/l_{2}}$$

where *p*_i_ is the radial pixel index, *p*_l_ is the physical pixel size, *q*_2_ is the distance from L2 to D, *l*_1_ is the distance from S to L1, and *l*_2_ is the distance from BS to L2. For simplicity, *q*_2_ is equal to the effective focal length (EFL) of L2, and *l*_1_ is equal to the ELF of L1, or slightly longer. *l*_2_ is the only free parameter, which changes the magnification of the projection onto the detector. For the example set-up shown in Figure 6b, *l*_1_ is 15 mm, *p*_l_ is 0.0048 mm, *q*_2_ is 25.43 mm. Therefore, to capture the entire 40° (this is the maximum scattering angle, based on arctan (*R*_L1_/*l*_1_), where *R*_L1_ is the radius of L1) captured scattering, within the detector 600 pixels across, *l*_2_ has to be equal to 92 mm. Moving the sample away to *l*_1_ = 25 mm, increases the *l*_2_ distances to 119 mm. The *q*-range is calibrated with a diffraction grating and has been determined to be within 1% of the expected value based on Equation (5). We note that this is a standard experimental design for benchtop static scattering systems [20]. We built up on that design to include intensity correlations as a function of the scattering angle, *θ*, and the spatial azimuthal angle, *ψ*.

In Figure 6b, an example benchtop SALS set-up is shown. The vertically polarized He Ne or diode laser power and beam can be adjusted using a neutral density filter (ND) and a pinhole (P), and *l*_1_ and *l*_2_ may be changed to optimize the *q* range of interest. To acquire data, the 2D scattering images are captured at 480 frames per second for 11 s. At optical wavelengths of 400–800 nm, the overall *q* range is 0.00035–0.035 nm^−1^. To resolve typical polymer dynamics (*D* ~ 2 × 10^−11^ m^2^/s), the temporal resolution only has to be 2–25,000 fps. At 500 fps, dynamics at *q* = 0.005 nm^−1^ (14° for a 625 nm laser) are easily determined. Therefore, using a camera operating at only 480 fps, polymer diffusion coefficients and gel dynamics can be determined by autocorrelating the intensity fluctuations as a function of *q*.

To obtain the dynamic information from SALS measurements, wave vector dependent autocorrelation function was firs1t computed from the images:

(6) $$G_{2}(q,\Delta t)=\frac{1}{t_{0}}\int_{0}^{t_{0}}I(t)I(t+\Delta t)d\Delta t$$

where *t*_0_ is the total time. *I*(*t* + Δ*t*) and *I*(*t*) are respectively the scattering intensity at future and present times. Since the desired dynamics are related to temporal intensity fluctuations, the normalized autocorrelation function was obtained by approximating the integral in Equation (6):

(7) $$g_{2}(q,\Delta t)=\frac{\frac{1}{n}\sum_{1}^{n}\left(I_{t+\Delta t}^{\prime }-I_{t}^{\prime }\right)}{\sigma_{I}^{2}}$$

where, $\;\sigma_{I}^{2}$ is the intensity variance. $\;I_{t+\Delta t}^{\prime }$ and $I_{t}^{\prime }$ are respectively the intensity fluctuation at future and present time. Rather than autocorrecting at every instant in time, $\;g_{2}\left(q,\Delta t\right)$ may be computed at exponentially spaced times to reduce the computational cost. To extract dynamics, autocorrelation functions are analyzed using a series of exponential decay. Two rate constants are extracted, *Γ*_1_ and *Γ*_2_, corresponding to network relaxations and inhomogeneities respectively.

### 4.4. DSALS Sample Preparation

The DSALS samples consist of two cleaned no.1 glass cover slips separated by a silicone spacer (~2 mm in width). The silicone spaces are cut to a rectangular shape ~2 inches in length and 0.5 inches in width. A circular 0.3-inch radius hole was then punched in the middle. The mold was etched with a Jelight (Irvine, CA, USA) UV-Ozone Model 24 cleaner for a 20-min to allow covalent bonding between the silicone mold and the PVA gel during crosslinking. A plastic round cover slip was placed at the bottom of the mold. The 2 wt% PVA solution and glutaraldehyde with the desired concentration are added into the mold by a pipette, and another plastic round cover was placed on the top of the mold to seal the sample to minimize water evaporation. The sample was then held for at least 12 h for the gelation to reach equilibrium before the DSALS measurement. Once gelation is complete, the plastic coverslips were removed without damaging the gel, and UV-Ozone etched-no.1 coverslips are placed on each side of the sample.

### 4.5. Stretching Experiments

To stretch the gels, the rectangular silicone mold with the gel sample was secured onto a homemade tensile stretching geometry using plate grips (See Supplementary Information). The plate grips ensure that consistent forces act on silicone mold when stretched. The silicone mold was then stretched 1.3 times. After stretching, a drop of filtered water was added to the surface of the gel, and covered by cleaned no.1 glass coverslips. The setup was allowed to reach equilibrium for ~20 min before the scattering experiments.

## Figures and Tables

**Table 1:** Comparison of dynamic techniques.

Technique	Contrast	*q*-Range	Sample Volume	1D or 2D
DSALS	Refractive index	0.00035–0.035 nm^−1^	~10–500 µL	1D
XPCS	Electron density	0.05–10 nm^−1^	~10–500 µL	2D
DLS	Refractive index	0.00–0.025 nm^−1^	~1–10 µL	2D

## Data Availability

The data that support findings of this study are available from the corresponding author upon reasonable request.

## Supplementary Materials

The following supporting information can be downloaded at: https://www.mdpi.com/article/10.3390/gels8070394/s1, Figure S1. PVA dynamics determined from DSALS and DLS. (a) DLS intensity correlation at varying θ angles. The black line is a double exponential fit to the data. (b) DSALS intensity correlations at varying q. The black line is a double exponential fit to the data; Figure S2. Comparison of PVA gel dynamics at different azimuthal angle ψ via DSALS. (a) DSALS intensity correlation at an azimuthal angle, ψ = 45°. The black line is a double exponential fit to the data. (b) The dominant fast relaxation rate *Γ* determined from the exponential fit vs. *q*^2^ as a function of azimuthal angle, ψ, determined from DSALS; Figure S3. Schematic of gel stretching sample holder. The PVA is covalently bound to the silicone mold (peach), such that the mold deformation is translated to the gel; Figure S4. Comparison of stretched PVA gel dynamics at different azimuthal angle ψ via DSALS. (a) *q* = 2.245. (b) *q* = 2.124.
