# Supplementary material for: Spatially-Resolved Network Dynamics of Poly(vinyl alcohol) Gels Measured with Dynamic Small Angle Light Scattering
Source: Gels. 2022 Jun 22;8(7):394. doi: 10.3390/gels8070394 (PMC9316599; doi:10.3390/gels8070394)
Supplement: Supplementary file 1 [file gels-08-00394-s001.zip › gels-1761748-supplementary.pdf]

Supplementary Materials

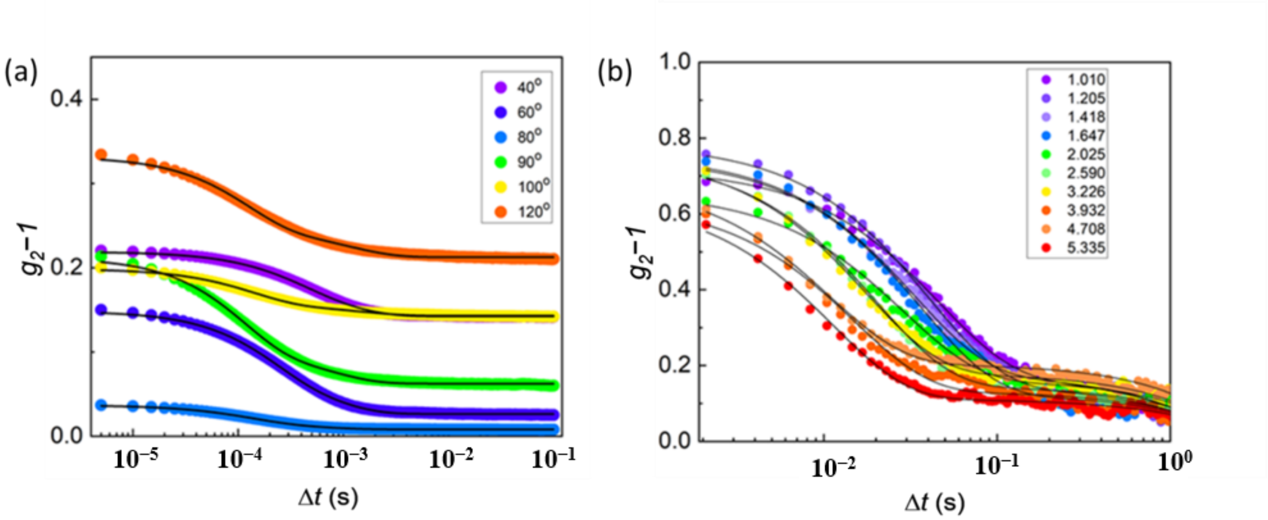

Figure S1. PVA dynamics determined from DSALS and DLS. (**a**) DLS intensity correlation at varying θ angles. The black line is a double exponential fit to the data. (**b**) DSALS intensity correlations at varying q. The black line is a double exponential fit to the data.

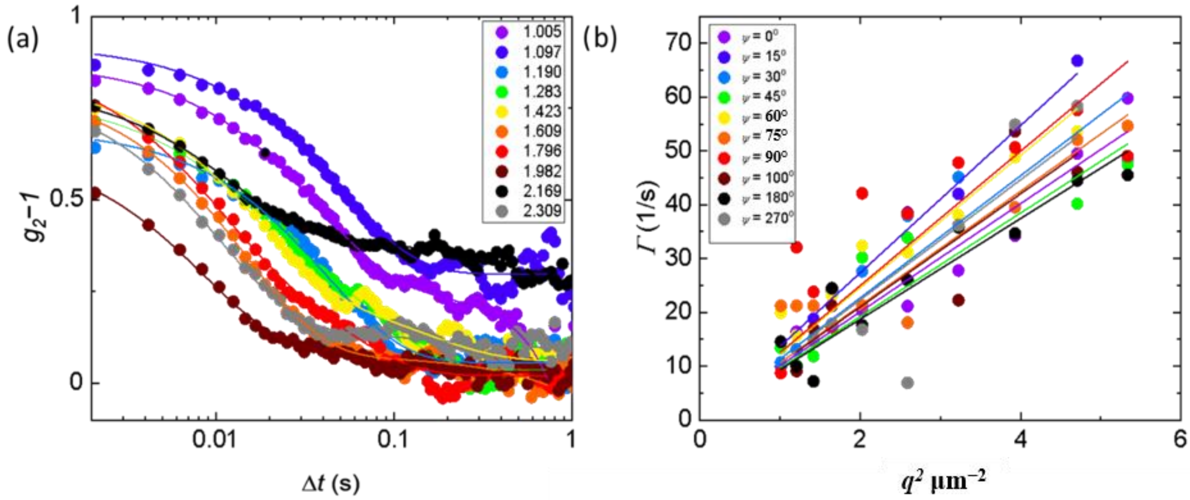

Figure S2. Comparison of PVA gel dynamics at different azimuthal angle ψ via DSALS. (**a**) DSALS intensity correlation at an azimuthal angle, $\psi = 45^o$. The black line is a double exponential fit to the data. (**b**) The dominant fast relaxation rate Γ determined from the exponential fit vs. q^2 as a function of azimuthal angle, ψ, determined from DSALS.

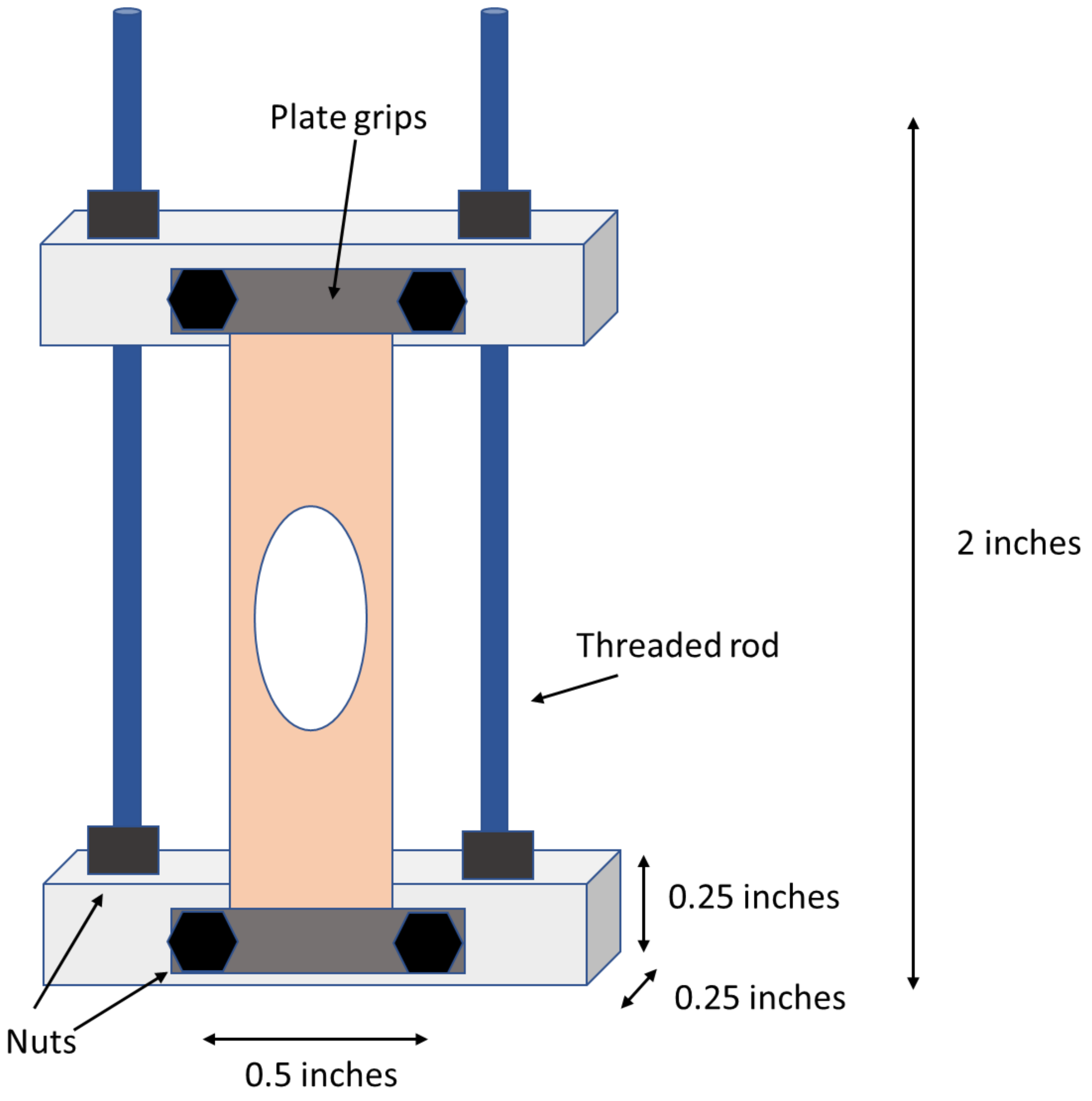

Figure S3. Schematic of gel stretching sample holder. The PVA is covalently bound to the silicone mold (peach), such that the mold deformation is translated to the gel.

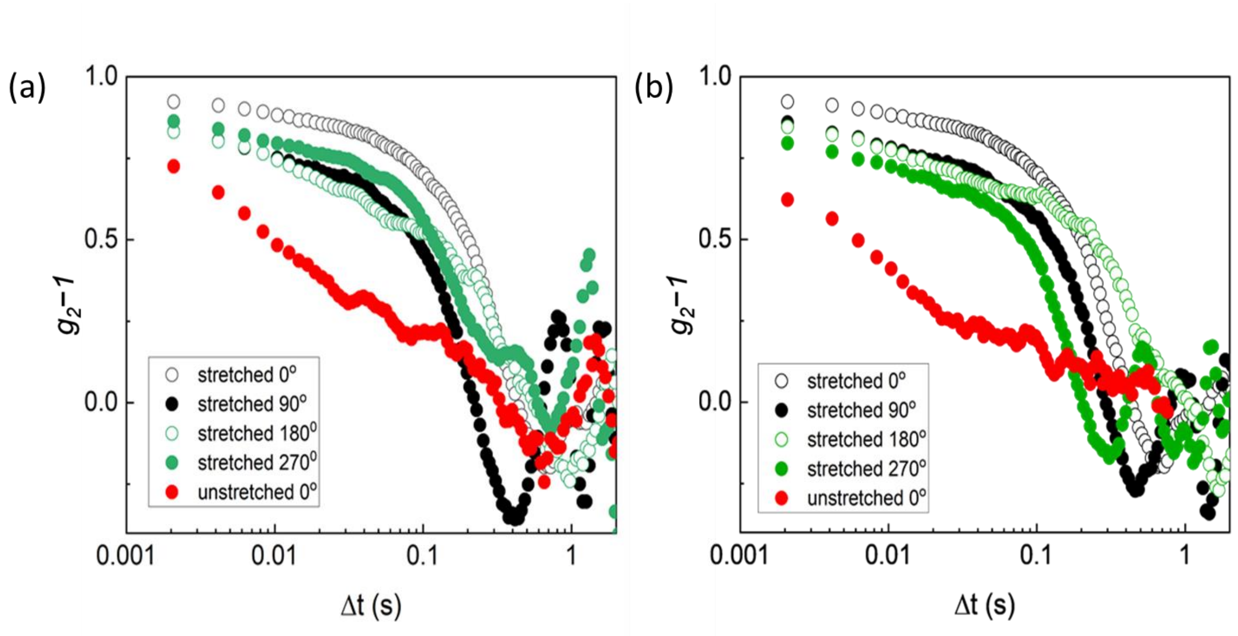

Figure S4. Comparison of stretched PVA gel dynamics at different azimuthal angle ψ via DSALS. (**a**) q = *2.245*. (**b**) q = *2.124*.
